# Supplementary figures and images for: Circulating CTRP9 correlates with the prevention of aortic calcification in renal allograft recipients
Source: PLoS One. 2020 Jan 16;15(1):e0226526. doi: 10.1371/journal.pone.0226526 (PMC6964899; doi:10.1371/journal.pone.0226526)

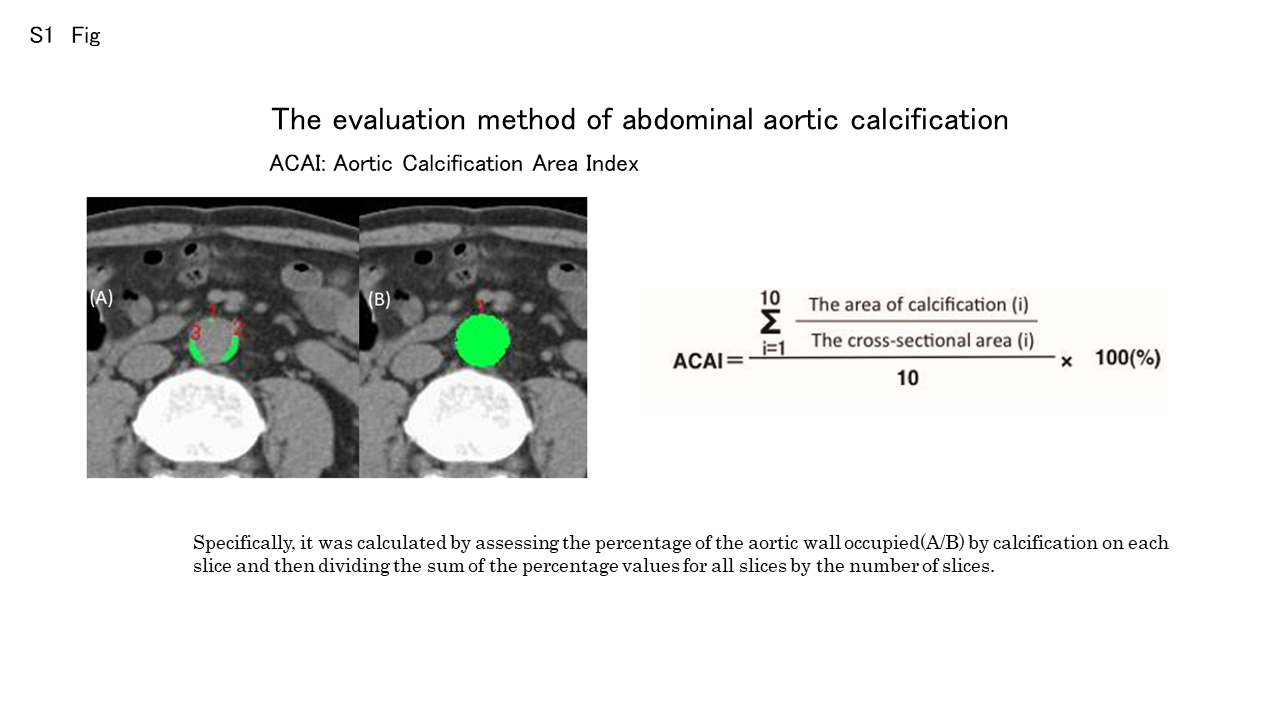

Supplement: S1 Fig — It was calculated by assessing the percentage of the aortic wall occupied by calcification on each slice and then dividing the sum of the percentage values for all slices by the number of slices. (TIF) [file pone.0226526.s001.TIF]

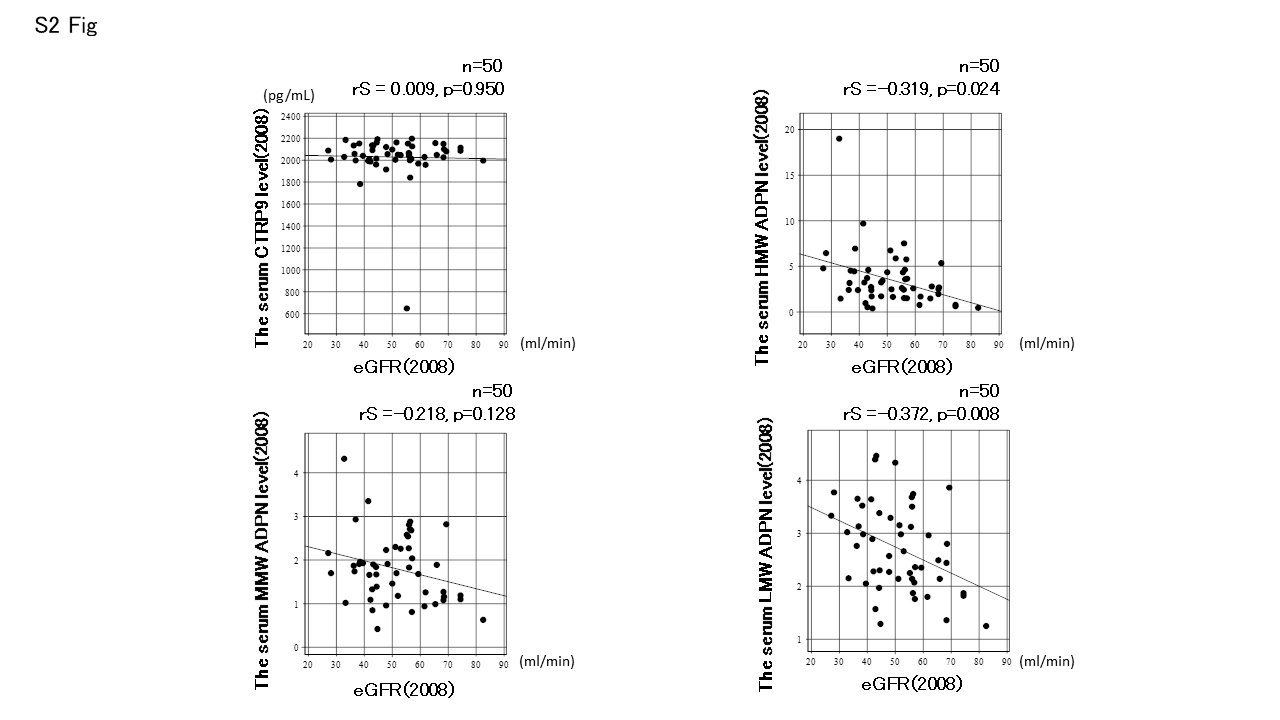

Supplement: S2 Fig — The CTRP9 and MMW-ADPN level was not significantly correlated with the eGFR (rS = -0.009, p = 0.950, n = 50, rS = -0.218, p = 0.128, n = 50, respectively), whereas the HMW-and LMW-ADPN levels were inversely correlated with the eGFR (rS = -0.319, p = 0.024, n = 50 and rS = -0.372, p = 0.008, n = 50, respectively), as previously reported. (TIF) [file pone.0226526.s002.TIF]

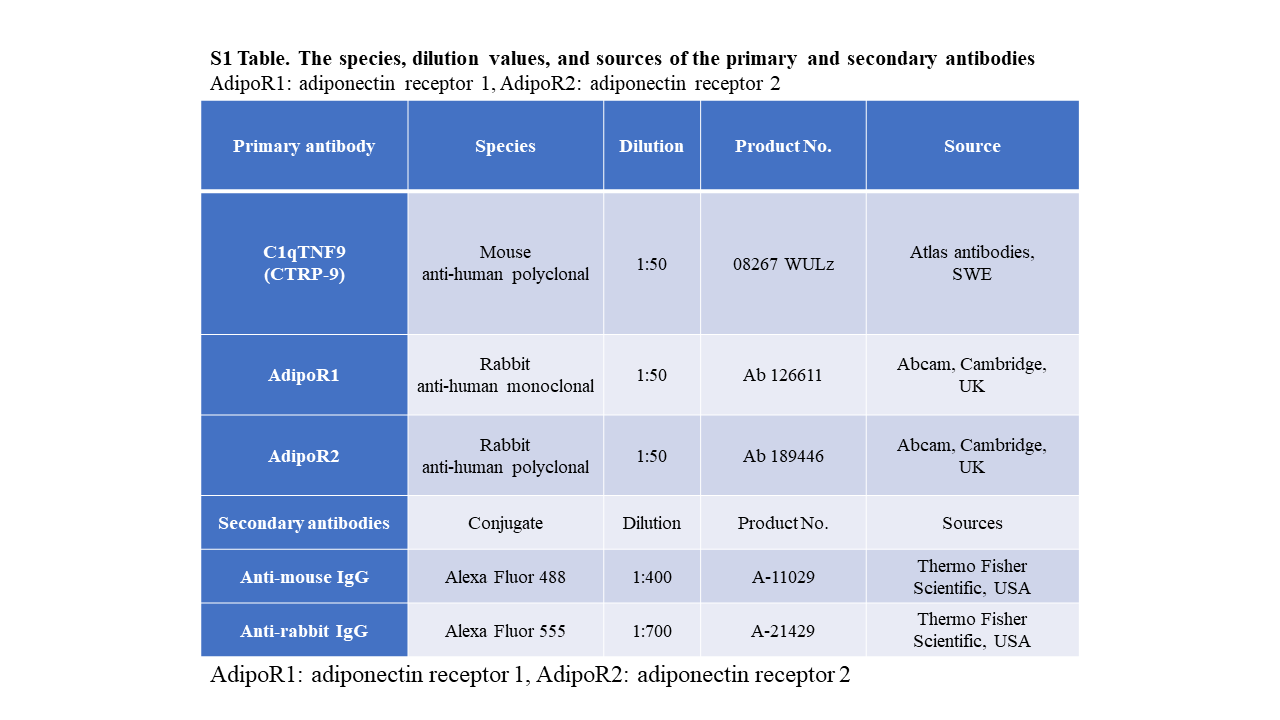

Supplement: S1 Table — (TIF) [file pone.0226526.s003.tif]

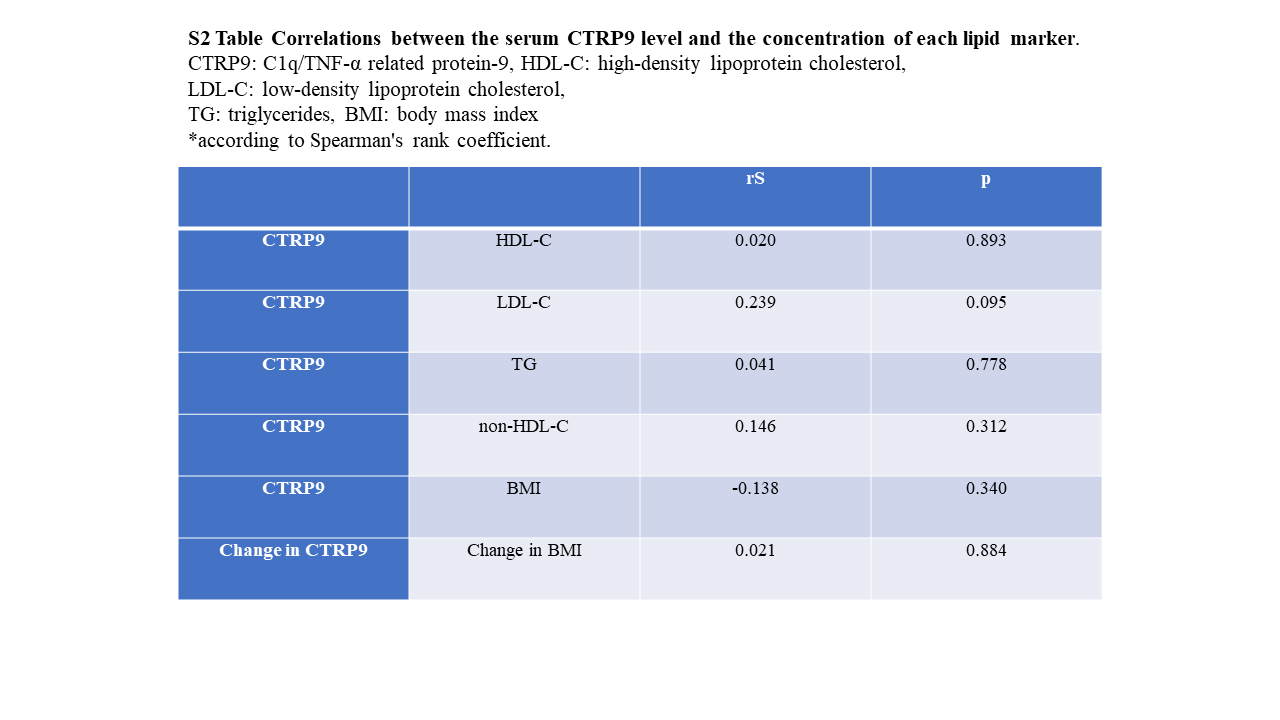

Supplement: S2 Table — (TIF) [file pone.0226526.s004.tif]
