# Supplementary material for: Circulating CTRP9 correlates with the prevention of aortic calcification in renal allograft recipients
Source: PLoS One. 2020 Jan 16;15(1):e0226526. doi: 10.1371/journal.pone.0226526 (PMC6964899; doi:10.1371/journal.pone.0226526)
Supplement: Attachment — Submitted filename: response to reviewers .docx [file pone.0226526.s006.docx]

Date: October16, 2019

Editor office “PLOS ONE”

Dear to Editor,

Thank you very much for your thoughtful comments.

We would like to re–submit the revised manuscript entitled “Circulating CTRP9 correlates with the prevention of aortic calcification in renal allograft recipients’’.

We are most grateful to you and the reviewers for the helpful comments on the original version of our manuscript.

We have taken all these comments into account and submit.

According to your reviewer’s comments, we improved our manuscripts as below.

The reviewer-1 comments:

This manuscript entitled　“Circulating CTRP9 correlates

with the prevention of aortic calcification in renal allograft recipients” written by Miyatake et al showed that the change in the serum CTRP9 concentration was positively correlated with the change in the serum HMW-ADPN concentration, and that multiple regression analysis showed that the change in the serum CTRP9 concentration was negatively associated with the change in ACAI. These findings are potentially interesting and useful data to understand the increased development of atherosclerotic　 lesions in renal allograft recipients. However, their data do not have the important confounding factors, such as high sensitive CRP or TNF−α. In addition, immuno-histological staining of Adipo R1 and R2 is quite poor images.

Comment #1:

As the authors mentioned in Discussion, the concentration of CTRP9 and HMW-ADPN is strongly associated with the inflammatory state. Thus, the authors should measure some inflammatory mediators like high-sensitive CRP, TNF−α, IL-6.

Response:

We think it difficult to measure the mediators such as high-sensitivity CRP, TNF−α, and IL-6, as you pointed out. One reason is that we thought they were not significantly different because we measured them in a stable state, and another reason is the limited amount of sample needed for measurement.

Comment #2:

In Figure 5, the staining of Adipo R1 and R2 looks non-specific. These data do not show the proof of co-localization between CTRP9 and Adipo R1. The authors should exhibit more clear stain. Or, Figure 5 should be removed.

Response:

We re-stained in another case for Adipo R1 and R2 IS (immunofluorescent staining), to confirm more specific staining. Hence, the conditions of reagents used for staining have been changed. As a result, more specific staining was obtained for CTRP9 and AdipoR1. A clearer colocalization was confirmed. We can send the newly obtained image in a manuscript here.

The reviewer-2 comments:

The manuscript is the 1st report on the negative correlation of circulation CTRP9 and aortic calcification in renal transplantation patients.

Comment #1:

Renal allograft IS (co-location of CTRP9 and AdipoR1) was not able

to lead the conclusion ‘CTRP9 and HMW-ADPN were suggested to prevent the progression of aortic calcification through AdipoR1. Because chronic rejection implicates the allograft vessels directly. To address this concern, animal allogeneic solid organ transplantation model (e.g. heart, kidney or liver) could be set and non-relevant artery (abdominal artery) should be stained for co-location to exclude the direct allorejection background.

Response:

In this study, the proof of CTRP9 and AdipoR1 collocation in renal allograft IS in this study was aimed to show that the CTRP9 expression site coincides with the localization of AdipoR1. As you pointed out, we think that this IS alone cannot prove the process of suppressing vascular calcification. In addition, animal experiments of organ transplantation models, such as your proposal, will be difficult to carry out at our facility.

Comment #2:

What’s the relationship of concentration of CTRP9 (and HMW-ADPN)

with ACAI at endpoint (2016)?

Response:

We couldn’t confirm significant correlation CTRP9 and HMW-ADPN concentrations with concurrent ACAI at the 2016 endpoint.

Comment #3:

How were the “concentration changes” calculated? This should be described in “Method” section.

Response:

We used the calculation method of concentration change is shown below.

*Change in CTRP9= {(serum CTRP9 level in 2008)- (serum CTRP9 level in 2016)}/8*

*Change in HMW ADPN= {(serum HMW-ADPN level in 2008)- (serum HMW-ADPN level in 2016)}/8*

*Change in ACAI= {(ACAI in 2008)- (ACAIin 2016)}/8*

The method for calculating the concentration change is shown in the Method section.

We will continue to make corrections centered on the above points and send the revised manuscript by the due date.

We thank for your advices.

Nobuhiko Miyatake, MD C/O Hitoshi Yokoyama, MD, PhD

Kanazawa Medical University School of Medicine

Department of Nephrology

1-1 Daigaku, Uchinada, Ishikawa 920-0293, JAPAN.

Tel: +81-76-218-8166

FAX: +81-76-286-2786

E-mail: nobuhiko@kanazawa-med.ac.jp
