# Supplementary material for: Circulating CTRP9 correlates with the prevention of aortic calcification in renal allograft recipients
Source: PLoS One. 2020 Jan 16;15(1):e0226526. doi: 10.1371/journal.pone.0226526 (PMC6964899; doi:10.1371/journal.pone.0226526)
Supplement: Attachment — Submitted filename: response to reviewers 20191126.docx [file pone.0226526.s007.docx]

Date: November 26, 2019

Editor office “PLOS ONE”

Dear to Editor,

Thank you very much for your thoughtful comments.

We would like to re–submit the revised manuscript entitled “Circulating CTRP9 correlates with the prevention of aortic calcification in renal allograft recipients’’.

We are most grateful to you and the reviewers for the helpful comments on the original version of our manuscript.

We have taken all these comments into account and submit.

According to your reviewer’s comments, we improved our manuscripts as below.

Comment #1:

Thank you very much for your thoughtful comment.

Unfortunately, this study was a retrospective study, then the collected samples were insufficient in some cases. Therefore, it was difficult to measure additionally and to compare with other suggested molecules such as high sensitivity CRP and TNF-α. We added the following sentence in limitation; 3) not using high accurate inflammatory markers such as sensitive CRP or TNF−α.

However, the concentration of CTRP9 or HMW-ADPN is also strongly associated with the inflammatory state as we mentioned in Discussion.

We hope our revision certified your comment.

We thank you again for your advice.

Nobuhiko Miyatake, MD C/O Hitoshi Yokoyama, MD, PhD

Kanazawa Medical University School of Medicine

Department of Nephrology

1-1 Daigaku, Uchinada, Ishikawa 920-0293, JAPAN.

Tel: +81-76-218-8166

FAX: +81-76-286-2786

E-mail: nobuhiko@kanazawa-med.ac.jp
